# Facultative and obligate diapause phenotypes in populations of the European spruce bark beetle *Ips typographus*

**DOI:** 10.1007/s10340-021-01416-w

**Published:** 2021-08-05

**Authors:** Martin Schebeck, Nina Dobart, Gregory J. Ragland, Axel Schopf, Christian Stauffer

**Affiliations:** 1 Department of Forest and Soil Sciences, University of Natural Resources and Life Sciences, Vienna, BOKU, Vienna, Austria; 2 Department of Integrative Biology, University of Colorado-Denver, Denver, CO USA

**Keywords:** Bark beetle, Dormancy, Voltinism, Life cycle regulation, Forest pest, Seasonality

## Abstract

The bark beetle *Ips typographus* is the most destructive insect pest in Norway spruce-dominated forests. Its potential to establish multiple generations per year (multivoltinism) is one major trait that makes this beetle a severe pest. *Ips typographus* enters diapause to adjust its life cycle to seasonally changing environments. Diapause is characterized by developmental and reproductive arrest; it prolongs generation time and thus affects voltinism. In *I. typographus* a facultative, photoperiod-regulated diapause in the adult stage has been described. In addition, the presence of an obligate, photoperiod-independent, diapause has been hypothesized. The diapause phenotype has important implications for *I. typographus* voltinism, as populations with obligate diapausing individuals would be univoltine. To test for the presence of different *I. typographus* diapause phenotypes, we exposed Central and Northern European individuals to a set of photoperiodic treatments. We used two ovarian traits (egg number and vitellarium size) that are associated with gonad development, to infer reproductive arrest and thus diapause. We found a distinct effect of photoperiod on ovarian development, with variable responses in Central and Northern European beetles. We observed obligate diapausing (independent of photoperiod) individuals in Northern Europe, and both facultative (photoperiod-regulated) as well as obligate diapausing individuals in Central Europe. Our results show within-species variation for diapause induction, an adaptation to match life cycles with seasonally fluctuating environmental conditions. As the diapause phenotype affects the potential number of generations per season, our data are the basis for assessing the risk of outbreaks of this destructive bark beetle.

**Supplementary Information:**

The online version contains supplementary material available at 10.1007/s10340-021-01416-w.

## Key Message

- Diapause, a common strategy to cope with adversity, affects insect seasonality and voltinism.
- *Ips typographus* expresses a facultative, photoperiod-regulated diapause.
- In addition, here we describe an obligate, photo-insensitive diapause.
- Facultative and obligate diapause phenotypes show geographic differences in Europe.
- Diapause affects the number of generations/year and is thus important for risk assessment.

## Introduction

Bark beetles are common insects in forest ecosystems (Hulcr et al. 2015; Raffa et al. 2015). The majority of these beetles live in dead and dying plants, and thus have an important ecological function as early decomposers. Some bark beetle species, however, are capable of colonizing healthy or moderately stressed hosts and are therefore important forest pests (Kirkendall et al. 2015). In Europe, *Ips typographus* (L.) (Coleoptera, Curculionidae, Scolytinae) is the most destructive pest in Norway spruce-dominated forests (Kausrud et al. 2012; Schelhaas et al. 2003; Wermelinger 2004). It shows extensive population outbreaks, which are usually triggered by abiotic disturbance events, e.g. storm, snowfall or drought, providing high amounts of suitable breeding material. This usually results in a rapid increase of beetle population densities, followed by severe Norway spruce mortality (Marini et al. 2017; Seidl et al. 2016; Stadelmann et al. 2014; Wermelinger 2004).

In addition, several intrinsic, species-specific traits account for the potential of *I. typographus* to efficiently colonize and kill host trees. For example, it has a high fecundity (Anderbrant 1990; Anderbrant and Löfqvist 1988), an efficient intraspecific chemical communication (Schlyter et al. 1987a; Schlyter et al. 1987b), associations with blue-stain fungi (Horntvedt et al. 1983; Lieutier et al. 2009) and it can establish multiple generations per year (Wermelinger 2004; Wermelinger et al. 2012). *Ips typographus* voltinism strongly varies across its geographic range. For instance, in Central Europe and low elevation habitats it usually produces two, sometimes three, generations per year (Baier et al. 2007; Wermelinger et al. 2012). In Northern Europe and high elevation sites only one generation is often established (Jönsson et al. 2009; Wermelinger 2004). Major parts of the beetle’s life history, like developmental rate or reproduction, are driven by ambient temperature (Wermelinger and Seifert 1998; Wermelinger and Seifert 1999). In addition, photoperiod is another important factor regulating the *I. typographus* life cycle, as it influences the induction of a reproductive diapause (Dolezal and Sehnal 2007; Schebeck et al. 2017; Schopf 1985; Schopf 1989; Schroeder and Dalin 2017).

Diapause is a common phenotype in insects that is induced during unfavourable seasons (Tauber and Tauber 1976; Tauber et al. 1986). It is characterized by a suppression of morphological development and reproduction, that is often accompanied by suppressed metabolic rate and enhanced resistance to external stressors (Denlinger 2002; Hahn and Denlinger 2011; Kostal 2006). In addition to an increased chance of survival during adverse periods, diapause prolongs generation time and thus affects phenology and voltinism (Danks 1987; Visser et al. 2010). Therefore, understanding the physiology and environmental sensitivity of diapause is not only important to understand seasonality and life cycle regulation of insects, it has also important implications for pest management as it influences the potential number of generations per year. Diapause is often not a fixed, or obligate element of a species’ life cycle; instead, induction of diapause is often mediated by environmental signals, e.g. photoperiod or thermoperiod (Denlinger 2002; Kostal 2006). Widely distributed species often exhibit marked clines in photoperiod-sensitivity of diapause induction or termination that translate into voltinism clines (Bradshaw et al. 2003; Paolucci et al. 2013; Tauber and Tauber 1972; Winterhalter and Mousseau 2007).

*Ips typographus* enters a reproductive diapause in the adult stage with photoperiod as main regulator (i.e. facultative, or environmentally inducible) (Dolezal and Sehnal 2007; Schebeck et al. 2017; Schopf 1985; Schopf 1989; Schroeder and Dalin 2017). Diapause induction is driven by short day lengths (Dolezal and Sehnal 2007; Schopf 1989; Schroeder and Dalin 2017), but high temperatures can override the photoperiodic effect and delay the onset of diapause (Dolezal and Sehnal 2007). The critical day length to induce diapause (i.e. day length at which 50% of a population entered diapause) varies across the species’ European range, to adjust reproduction, development and stress resistance to local conditions. The photoperiodic cue is generally perceived in late summer/early fall (Dolezal and Sehnal 2007; Schopf 1989; Schroeder and Dalin 2017). Diapause is terminated by low temperatures in mid-winter and followed by a post-diapause quiescence until favourable conditions prevail the following spring (Dolezal and Sehnal 2007).

In most populations, the timing of diapause induction affects the voltinism of *I. typographus*. The beetles continue to develop in successive generations until individuals perceive the photoperiodic cue for diapause induction (Annila 1969; Dolezal and Sehnal 2007; Schopf 1985; Schopf 1989; Schroeder and Dalin 2017). Previous findings, however, reported that certain European populations established only one generation per year, although conditions, i.e. temperature and day length, would be favourable to continue reproduction and morphogenesis (Annila 1969; Dworschak 2013; Netherer 2003). In addition, some portion of individuals within Northern European populations were in diapause, even when they were exposed to extreme long day lengths (Dolezal and Sehnal 2007; Schroeder and Dalin 2017). Therefore, some authors hypothesized that some populations of *I. typographus* might enter diapause independent of a photoperiodic induction, and thus have an obligate diapause phenotype (Schebeck et al. 2017; Schroeder and Dalin 2017). However, robust quantitative evidence for obligate diapause has not been documented previously.

Here, we test for differences in the photoperiodic sensitivity of diapause induction and for the presence of an obligate diapause among Central and Northern European populations of *I. typographus*. We exposed beetles to different photoperiods under laboratory conditions and used two traits associated with gonad development, egg numbers and ovary size, to assess whether females entered reproductive arrest, and thus diapause. We hypothesize that an obligate diapause phenotype would rather be found in Northern Europe and result in univoltine populations, an adaptation to environments with short favourable seasons. These observations would have important implications to understand outbreak patterns of the most destructive pest of European spruce forests and unravel a long-standing question about *I. typographus* life history.

## Materials and methods

### Insect collection

To study the occurrence of *I. typographus* diapause phenotypes in different habitats, individuals from three geographic locations were sampled: one Central European, low elevation site (LOW; Prinzersdorf, Austria; 48°22′N, 15°48′E; 350 m a.s.l.), one Central European, high elevation location (HIGH; Gesäuse, Austria; 47°35′N, 14°38′E; 1500 m a.s.l.) and one Northern European, Scandinavian site (NORTH; Vindeln, Sweden; 64°12′N, 19°43′E; 300 m a.s.l.). Beetles of the overwintered, i.e. parental, generation were randomly collected from freshly colonized Norway spruce. This allowed us to study diapause expression with reproductively active *I. typographus*, all of the same phenological state. Live beetles were placed on spruce phloem and transferred to the laboratory for experimental trials.

### Experimental setup

A standard procedure to evaluate diapause expression (i.e. whether or not diapause has been induced) in *I. typographus* was applied (Dolezal and Sehnal 2007; Schroeder and Dalin 2017). One-hundred unsexed adults were put in insect cages with one log of Norway spruce (~60 cm length, ~25 cm diameter), with two independent logs per experimental trial. To initiate colonization of the log and brood establishment, beetles were first exposed to 25 °C and a photoperiod of 16 h light : 8 h dark for ten days. After males colonized a log and excavated a mating chamber, females joined them, mated and laid eggs. To test for the presence of an obligate diapause phenotype, diapause expression of this offspring generation developing in the laboratory (i.e. the first generation) under different conditions was evaluated.

On day 11, logs were transferred to various photoperiodic conditions. To assess diapause expression of *I. typographus* from LOW, HIGH and NORTH at different day lengths, three photoperiodic treatments were applied: Beetles from all three locations were exposed to long-day (LD, light (l) : dark (d) 16:8 h) and short-day (SD, l : d 8:16 h) conditions. In addition, extra-long-day (ELD, l : d 23:1 h) conditions were applied with NORTH individuals, to assess their response to longer day lengths in their natural habitat (maximum sunlight during summer ~21 h, plus civil twilight; www.shmi.se). Experiments were performed in incubators at constant temperatures of 20 °C, as the effect of photoperiod on diapause induction is overridden at temperatures above 23 °C (Dolezal and Sehnal 2007). All experimental trials were terminated after 80 days, as the development of one generation (from the egg to the end of the maturation feeding of young adults) at 20 °C lasts on average ~75 days (Wermelinger and Seifert 1998). Diapause expression of *I. typographus* was evaluated after individuals finished their development and left logs, or after the end of experiments when beetles remained in the bark (details see below).

### Measurements to evaluate diapause expression

We measured two characteristics of ovarian development to assess whether females were reproductively active or if they had entered reproductive arrest. The former phenotype suggests non-diapause development, while the latter suggests diapause induction. First, we counted the total number of eggs in the vitellarium (basal part of the ovariole) for each of the four ovarioles (Merker and Wild 1954), then calculated the mean number of eggs per ovariole per female. Second, we calculated the mean relative length of the vitellarium (vitellarium length [mm] : total ovariole length [mm]) for each of the four ovarioles for each female. Measurements of ovary size were performed with *IC Measure* (www.theimagingsource.de).

To infer effects of diapause expression on the life history of *I. typographus,* we also accounted for the emergence behaviour of young adults developing at SD, LD and ELD treatments. Generally, non-diapausing young adults emerge to establish a new generation, whereas diapausing beetles prepare for hibernation (either in the bark, i.e. no emergence, or in the litter, i.e. emergence) (Annila 1969). Thus, the two ovary traits of emerged and non-emerged females were evaluated, to assess their reproductive/diapause status. During the main phase of emergence from logs, about 25 females per experimental condition were collected daily and dissected. Likewise, on average 25 non-emerged females were randomly selected from their breeding systems (with only one individual per system) by de-barking the logs after the end of experiments and dissected as above (overview on sample sizes see Supplementary Information 1).

Distinguishing diapausing from non-diapausing females requires that we clearly define the ovarian development phenotypes (see above) for beetles known to be not in diapause. We thus established control cohorts of beetles in which diapause was induced and then terminated under conditions known to produce reproductively active, post-diapause females (Dolezal and Sehnal 2007). After beetles from all locations were exposed to SD and 20 °C for 80 days (individuals entered diapause), logs were exposed to chilling conditions at 5 °C for 90 days at SD (to terminate diapause), and then to LD and 20 °C (modified after (Dolezal and Sehnal 2007)). Subsequently, about 25 emerged females during peak emergence from logs per location were dissected and measured for the same two ovary traits described above.

### Data analyses

To assess whether *I. typographus* from LOW, HIGH and NORTH, developing under different photoperiodic conditions, form distinct clusters, the mean number of eggs per ovariole and the mean relative vitellarium length were visualized in a biplot. Subsequently, for each of the three locations the two ovarian traits of emerged and non-emerged females, developing at SD, LD and ELD, were compared to distinguish diapausing from non-diapausing individuals, with data of post-diapause females as control group for non-diapause development.

Before comparing the two ovarian traits among these groups, normal distribution of data was evaluated using a Shapiro–Wilk normality test. Normally distributed datasets were further analysed using an ANOVA. In case of variance homogeneity—evaluated by a Levene test in the R package *car* (Fox and Weisberg 2019)—ovarian traits among groups were subsequently compared applying Tukey’s HSD post hoc test. If the Levene test showed significant results (*p* < 0.05) a Welch test, with Games–Howell post hoc tests (R package *userfriendlyscience* (Peters 2018)), was applied. Non-normally distributed datasets were analysed using a Kruskal–Wallis test, with a Dunn test (with adjusted p-values applying the Benjamini–Hochberg method) as post hoc tests. All analyses were performed in R, version 4.0.2 (RCoreTeam 2020), and results were visualized using the packages *ggplot2* (Wickham 2016) and *ggthemes* (Arnold et al. 2021). Raw data of egg numbers and vitellarium size are provided in Supplementary Information 4.

## Results

### Beetles from Northern Europe suppressed ovarian development regardless of photoperiodic condition

**Fig. 1:**
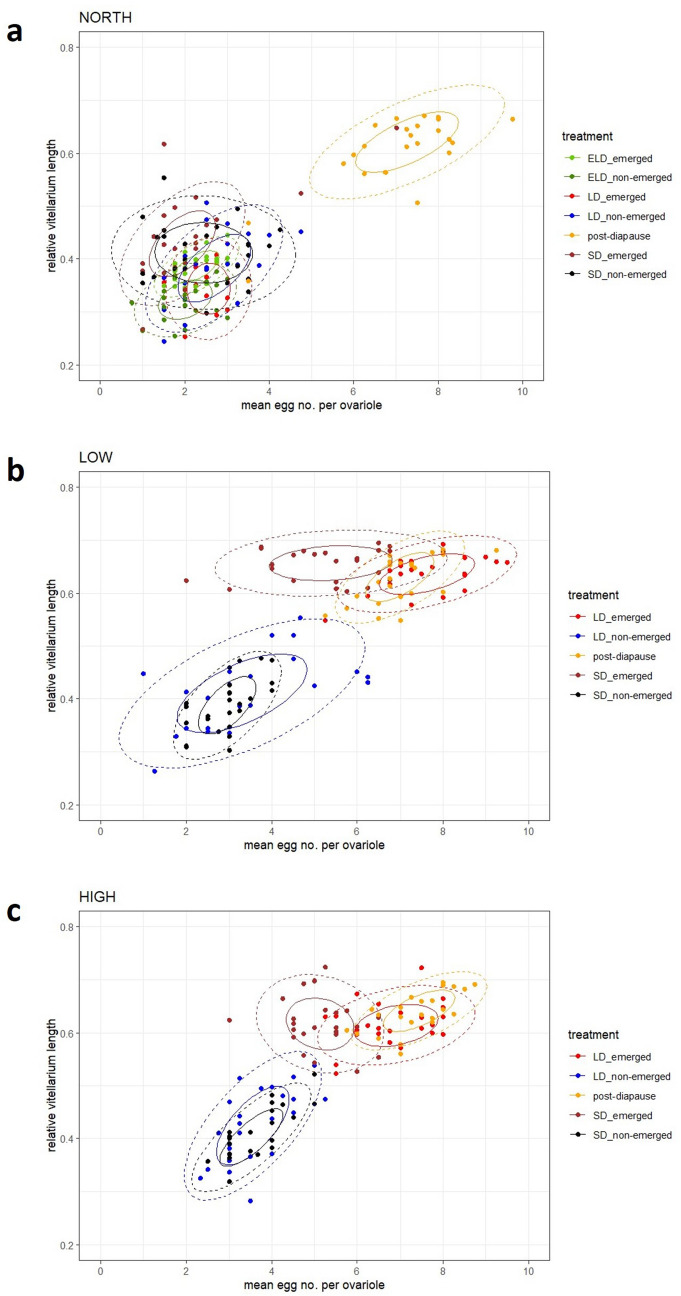
Grouping of emerged and non-emerged *Ips typographus* females by mean egg numbers and relative vitellarium length, developing at different photoperiodic conditions. ELD = extra-long-day (l : d 23 h : 1 h); LD = long-day (l : d 16 h : 8 h); SD = short-day (l : d 8 h : 16 h); post-diapause = development at diapause inducing and termination conditions, with subsequent transfer to favourable conditions. Solid line = 50th percentile of distribution of data. Dashed line = 90th percentile of distribution of data. **a** NORTH = Northern Europe, Scandinavia. **b** LOW = Central Europe, low elevation. **c** HIGH = Central Europe, high elevation

Visualizing the mean number of eggs per ovariole and the mean relative vitellarium length in a biplot revealed that NORTH females clearly clustered by experimental treatment. Post-diapause females were separated from emerged and non-emerged females developing at SD, LD and ELD. The 90th percentile around the centroids of the bivariate distributions for the two ovarian traits for post-diapause females did not overlap with the 90th percentile of beetles developing at all other experimental treatments, which showed strong overlaps among each other (Fig. 1a).

**Fig. 2:**
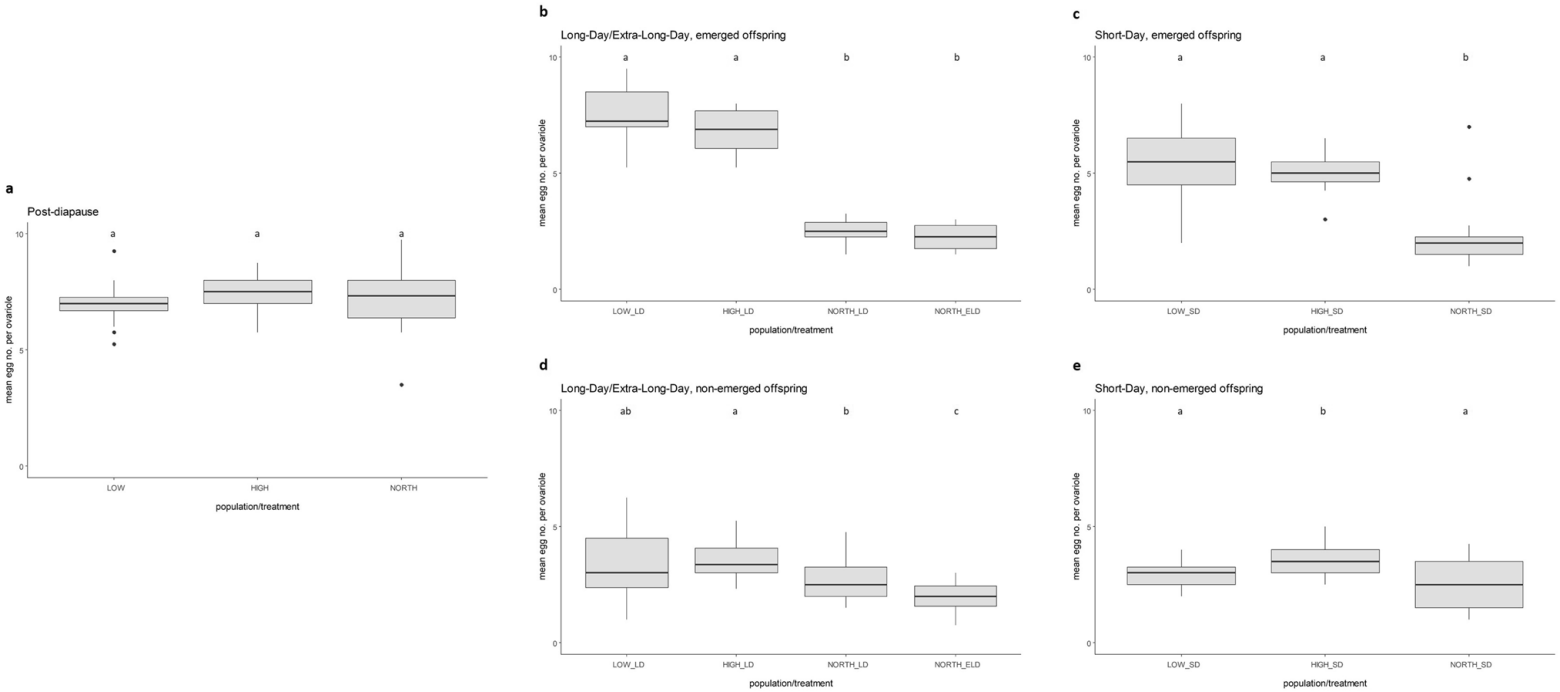
Mean number of eggs per ovariole in *Ips typographus* females developing at different experimental conditions. LOW = Central Europe, low elevation; HIGH = Central Europe, high elevation; NORTH = Northern Europe, Scandinavia. ELD = extra-long-day (l : d 23 h : 1 h); LD = long-day (l : d 16 h : 8 h); SD = short-day (l :  d 8 h : 16 h). **a** Post-diapause females from LOW, HIGH and NORTH. **b** Emerged females at LD and ELD. **c** Emerged females at SD. **d** Non-emerged females at LD and ELD. **e** Non-emerged females at SD. Different lower case letters indicate significant differences (*p* < 0.05)

The mean number of eggs of post-diapause females was significantly higher than of individuals exposed to all other conditions (*p* < 0.001, Kruskal–Wallis test, Dunn post hoc test), which suggests suppression of ovarian development at SD, LD and ELD, although a few outliers are observed (Figs. 1, 2). Emerged NORTH females developing at both LD and ELD conditions had low mean egg numbers (2.52 and 2.26 eggs/ovariole, respectively) which did not differ significantly (*p* = 0.552, Kruskal–Wallis test, Dunn post hoc test). Non-emerged females under LD and ELD had similar mean egg numbers (2.70 and 2.01 eggs/ovariole, respectively), which differed significantly (*p* = 0.021, Kruskal–Wallis test, Dunn post hoc test). Under SD conditions mean egg numbers were in the same range and not significantly different among each other (emerged females: 2.16 eggs/ovariole, non-emerged females: 2.43 eggs/ovariole; *p* = 0.226, Kruskal–Wallis test, Dunn post hoc test; Supplementary Information 1, further details on significant differences are provided in Supplementary Information 2 and Supplementary Information 3).

**Fig. 3:**
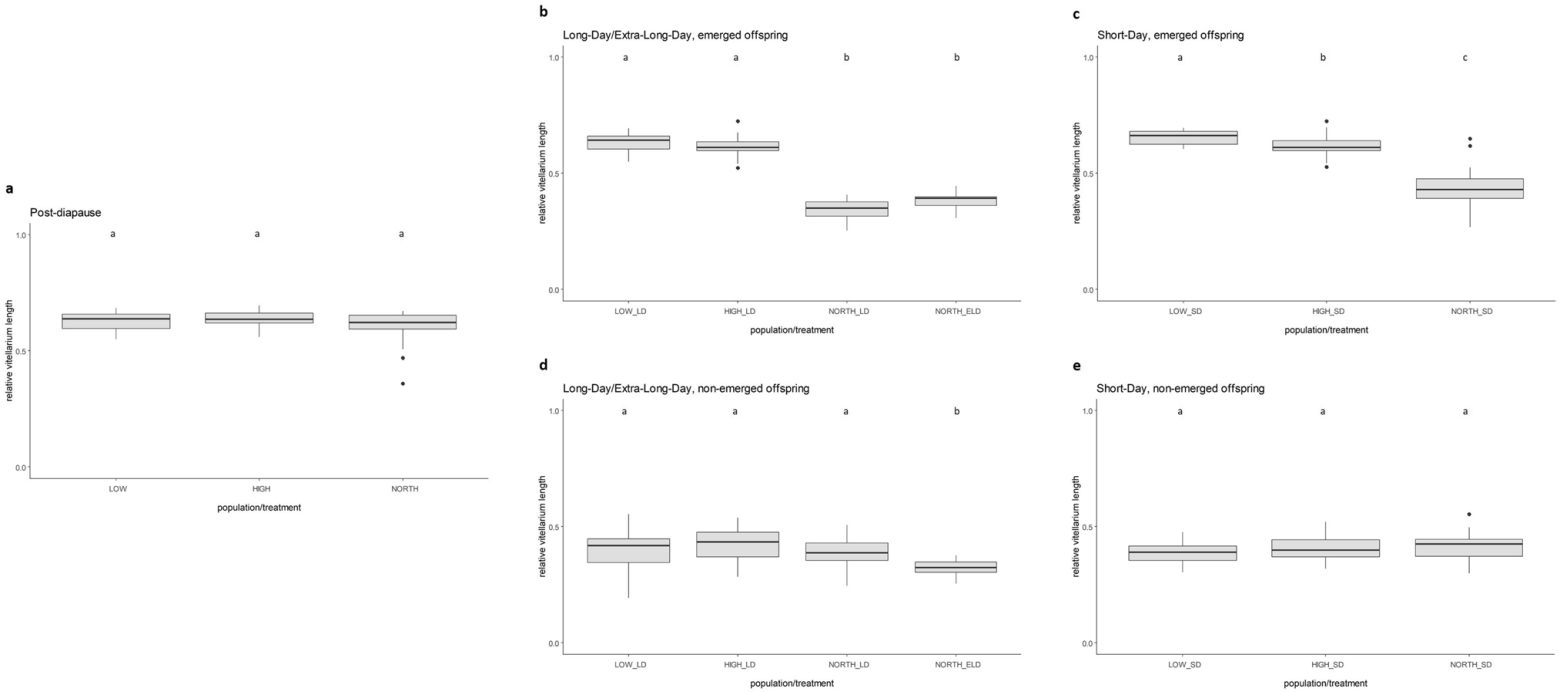
Mean relative vitellarium length of *Ips typographus* females developing at different experimental conditions. LOW = Central Europe, low elevation; HIGH = Central Europe, high elevation; NORTH = Northern Europe, Scandinavia. ELD = extra-long-day (l : d 23 h : 1 h); LD = long-day (l : d 16 h : 8 h); SD = short-day (l : d 8 h : 16 h). **a** Post-diapause females from LOW, HIGH and NORTH. **b** Emerged females at LD and ELD. **c** Emerged females at SD. **d** Non-emerged females at LD and ELD. **e** Non-emerged females at SD. Different lower case letters indicate significant differences (*p* < 0.05)

The mean relative vitellarium length of post-diapause individuals was also significantly longer as compared to the individuals that developed at all other treatments (*p* < 0.003, Kruskal–Wallis test, Dunn post hoc test). Individuals that developed at LD and ELD and emerged from logs had a similar relative vitellarium length, which did not differ significantly from each other (*p* = 0.335, Kruskal–Wallis test, Dunn post hoc test, Fig. 3). Both non-emerged females at LD and ELD had similar average relative vitellarium lengths (*p* = 0.001, Kruskal–Wallis test, Dunn post hoc test, Fig. 3). The mean relative vitellarium length of emerged and non-emerged individuals exposed to SD conditions was in the same range, with no significant differences (*p* = 0.328, Kruskal–Wallis test, Dunn post hoc test). Further, details on mean values and significant differences are provided in Supplementary Information 1, Supplementary Information 2 and Supplementary Information 3.

### Non-emerged beetles from Central Europe suppressed ovarian development at short days

Females from the two Central European locations that developed under SD conditions and did not emerge from logs showed similar mean egg numbers per ovariole (LOW: 2.95, HIGH: 3.58; *p* = 0.023, Kruskal–Wallis test, Dunn post hoc test, Fig. 2). The mean relative vitellarium length of these females was 0.39 in LOW and 0.41 in HIGH, which did not differ significantly from each other (*p* = 0.246, ANOVA, Fig. 3). These results on egg numbers and vitellarium size indicate a suppression of ovarian development in non-emerged SD beetles, as they were significantly different from Central European post-diapause females and emerged LD individuals (*p* < 0.001, Kruskal–Wallis test, Dunn post hoc test; details see Supplementary Information 3).

This was supported by the biplot results of LOW and HIGH where non-emerged SD females were clearly separated from reproductively active, post-diapause females, as the 90th percentile around the centroids of those groups in both populations did not overlap (Fig. 1b, c). Similarly, the relative vitellarium length of post-diapause females from both populations was significantly longer as compared to non-emerged SD individuals (*p* < 0.001, Kruskal–Wallis test, Dunn post hoc test; details see Supplementary Information 3).

### Beetles from Central Europe showed a variable response to long-day conditions

Emerged LD females from LOW had mean egg numbers of 7.61, emerged LD individuals from HIGH had a mean value of 6.85 eggs per ovariole, which did not differ significantly (*p* = 0.180, Kruskal–Wallis test, Dunn post hoc test, Fig. 2b). These numbers were in the same range compared to post-diapause females from the respective population, with no significant differences (LOW: *p* = 0.368, HIGH: *p* = 0.369, Kruskal–Wallis test, Dunn post hoc test, Fig. 2), suggesting no suppression of ovarian development in emerged LD females.

The relative vitellarium lengths of emerged individuals at LD from LOW and HIGH were in the same range and not significantly different (*p* = 0.297, Kruskal–Wallis test, Dunn post hoc test, Fig. 3). They had similar values as compared to post-diapause females from the respective population, also showing no significant differences (LOW: *p* = 0.906, HIGH: *p* = 0.297, Kruskal–Wallis test, Dunn post hoc test). These findings were supported by our biplot analysis where post-diapause females and emerged LD females in both LOW and HIGH showed strong overlaps among each other (Fig. 1 b, c).

A different pattern of ovarian development was found in LD females that did not emerge from logs. In LOW and HIGH on average 3.33 and 3.60 eggs per ovariole, respectively, were observed, which did not differ significantly (*p* = 0.132, Kruskal–Wallis test, Dunn post hoc test, Fig. 2). Similarly, the relative vitellarium length of these females did not show significant differences (*p* = 0.816, Kruskal–Wallis test, Dunn post hoc test, Fig. 3). Moreover, these females formed clusters with non-emerged SD individuals in the biplot analysis (Fig. 1b, c), suggesting a suppression of ovarian development when females remained in logs, even under LD photoperiods.

### Central European beetles can develop reproductively active ovaries under short-day conditions

We also observed Central European beetles that were exposed to SD conditions and emerged from logs. Females from LOW and HIGH had an average of about five eggs per ovariole, showing no significant differences among each other (*p* = 0.604, Kruskal–Wallis test, Dunn post hoc test, Fig. 2). The average relative vitellarium length of LOW females was 0.65 and that of HIGH individuals was 0.62, which differed significantly (*p* = 0.044, Kruskal–Wallis test, Dunn post hoc test, Fig. 3). The mean egg numbers of these females showed intermediate values between reproductively active, post-diapause individuals (and emerged LD individuals from Central Europe) and non-emerged SD females with suppressed ovary development (Fig. 2). However, the data on the relative vitellarium lengths were in the same range as in reproductively active individuals (Fig. 3, Supplementary Information 1; details on significant differences are provided in Supplementary Information 2 and Supplementary Information 3). Moreover, the biplot of the population LOW showed that the 90th percentile around the centroids of emerged LD and SD as well as post-diapause individuals were overlapping among each other, but were separated from the group consisting of non-emerged LD and non-emerged SD females (Fig. 1b). A similar pattern was observed in the biplot of the population HIGH, although a slight overlap among non-emerged LD and emerged SD females occurred (Fig. 1c).

## Discussion

### Gonad development and diapause in European *I. typographus*

The use of two ovarian traits, egg numbers and vitellarium size, helped to evaluate the reproductive status of *I. typographus* females and to infer whether diapause was induced or not. Data of post-diapause beetles provided information on reproductively active (= non-diapausing) individuals, which were subsequently used as control groups for comparisons with females developing at various photoperiodic treatments. Interestingly, results of egg numbers and vitellarium size of post-diapause females showed similar values across all locations, suggesting a comparable reproductive potential in individuals from different origin.

In Northern European *I. typographus* ovarian development of first-generation beetles was suppressed regardless of photoperiodic treatment, although a small number of outliers was observed. Females exposed to SD, LD and even ELD (either emerged or non-emerged) had fewer eggs in their ovarioles and shorter vitellarium lengths as compared to post-diapause individuals. This was corroborated by their clustering in the biplot analysis where post-diapause females were clearly separated from individuals developing at all other photoperiods. Moreover, values of the two ovary traits of Northern females from SD, LD and ELD treatments were in the same range as compared to Central European individuals with suppressed gonad development, e.g. non-emerged SD females. This indicates that first-generation females from Northern Europe entered diapause even under favourable environmental conditions, i.e. extreme long photoperiods. Comparing these results with the ovary development of NORTH post-diapause females shows that first-generation beetles from this population have to experience a chilling period, i.e. winter conditions in the field, to terminate diapause before becoming reproductively active, which suggests the expression of an obligate diapause. The existence of this phenotype has been hypothesized (Schebeck et al. 2017; Schroeder and Dalin 2017). Our detailed data on ovary development provide evidence for this hypothesis, a long-standing question in *I. typographus* life history.

Schroeder and Dalin (2017) described a critical day length of ~19 hours and a diapause incidence of about 20% at a day length of 23.5 hours for *I. typographus* from Northern Sweden (i.e. the same region as our NORTH population). Dolezal and Sehnal (2007), however, described a diapause incidence of 100% when exposing beetles from Southern Sweden to photoperiodic conditions of 18 : 6 h. Our results on egg numbers and vitellarium size clearly show that nearly all NORTH individuals were in diapause at all photoperiodic conditions, which illustrate a high incidence of obligate diapausing individuals in Northern Europe.

Similarly, ovary development was suppressed in Central European females that developed under SD photoperiods and remained in their breeding systems. Both egg numbers and vitellarium lengths were on average significantly smaller as compared to post-diapause females and emerged LD individuals, clearly indicating that individuals had entered diapause. These results support previous findings on diapause induction by short day length (Dolezal and Sehnal 2007; Schopf 1989).

In contrast, Central European beetles from LOW and HIGH that developed under LD and emerged from logs showed no suppression of gonad development, and thus no diapause expression. The values of the two ovarian traits were in the same range as in post-diapause individuals, corroborated by forming distinct clusters in the biplot analyses. This is in line with previous work where a critical day length for diapause induction of Central European *I. typographus* of <15 hours was described (Dolezal and Sehnal 2007; Schopf 1989), indicating a facultative diapause phenotype.

Interestingly, we also found females that were exposed to LD and remained in their breeding systems. These individuals showed ovarian traits that reflect a suppression of gonad development and therefore diapause expression, even under favourable photoperiodic conditions, suggesting the presence of obligate diapause phenotypes also in lower latitudes. These findings suggest genetic polymorphism in Central European *I. typographus* with different responses to diapause-inducing, photoperiodic cues. Our results might explain previous field observations where *I. typographus* was reproductively inactive although permissive temperature and photoperiodic conditions prevailed (Dworschak 2013; Netherer 2003).

Although developing at photoperiodic conditions below the critical day length, emerging *I. typographus* under SD conditions from Central European sites showed also ovarian traits reflecting no suppression of gonad development, i.e. no diapause. Despite having fewer eggs in their ovarioles than emerged LD females and post-diapause females, their vitellarium length showed values similar to non-diapausing and post-diapause beetles. Moreover, these emerged SD individuals clustered with post-diapause and emerged LD females in the biplot. These results suggest genetic polymorphism towards photoperiodic cues within populations, however, with lower egg numbers under short day lengths.

Taken together, we found clear differences in diapause expression and photoperiodic response in European *I. typographus* among geographically distinct populations. The presence of two diapause phenotypes, i.e. facultative and obligate, within this bark beetle species has been hypothesized previously (Schebeck et al. 2017; Schroeder and Dalin 2017). Our data show that *I. typographus* expresses a facultative (photoperiod-regulated) diapause, corroborating previous findings (Dolezal and Sehnal 2007; Schopf 1989; Schroeder and Dalin 2017). Moreover, we found Central and Northern European individuals expressing diapause, even at suitable photoperiodic conditions, i.e. LD and ELD. Therefore, we propose that *I. typographus* does not only enter a facultative, photoperiod-mediated diapause. It also expresses an obligate diapause in both Central and Northern European populations, where individuals became insensitive to photoperiodic cues that are experienced under natural conditions. Future studies should focus on diapause expression of *I. typographus* from different origin, to increase our knowledge on the occurrence and geographic distribution of facultative and obligate diapause phenotypes in European populations.

### *Ips typographus* diapause and its effects on seasonality and voltinism

The presence of two diapause phenotypes, i.e. facultative and obligate, has major implications for the phenology and voltinism of *I. typographus*. Populations with facultative diapausing individuals can produce new offspring generations until diapause is induced by short-day photoperiods in late summer/early fall (Dolezal and Sehnal 2007; Schopf 1989; Schroeder and Dalin 2017). Therefore, populations with facultative diapausing individuals have the potential to establish multiple generations per year, i.e. multivoltinism, assumed suitable temperature conditions prevail (Baier et al. 2007; Jakoby et al. 2019; Jönsson et al. 2009; Wermelinger et al. 2012). Populations with obligate diapausing *I. typographus*, however, can produce only one generation per year (univoltinism) as individuals enter reproductive and developmental arrest as part of their life cycle. In these univoltine populations diapause has to be terminated by low winter temperatures (Dolezal and Sehnal 2007) to resume reproduction and morphogenesis the following spring.

A few individuals in the Northern European population, however, had higher egg numbers similar to Central European females (e.g. outliers in Figs. 1, 2). They seem to have some capacity to develop successive generations in one season, reflecting a certain potential for bivoltinism in this region of the species’ range.

Expressing an obligate diapause can result in a trade-off. The reproductive outcome of *I. typographus* might be smaller when only one generation per year is produced, however, individuals that refrain from establishing a second generation can increase their overwinter survival. The favourable season in higher latitudes is generally shorter than in the South and *I. typographus* leaves its breeding system to search for a suitable overwintering site already in summer or stays in the bark to prepare for hibernation (Annila 1969; Schebeck et al. 2017). *Ips typographus* survives harsh winters only as an adult (Stefkova et al. 2017). Thus, beetles that express an obligate diapause refrain from producing a second generation that might not reach the adult stage before the onset of the cold season, resulting in a high risk of pre-imaginal overwintering mortality. Instead *I. typographus* likely invests resources for physiological and behavioural preparations (Kostal et al. 2011; Kostal et al. 2007) for a safe overwintering of adults and resumes reproduction the following year.

In upcoming research, diapause development of *I. typographus* should be studied from numerous populations across its entire range. This would provide important data on the occurrence of obligate and facultative diapausing individuals*,* and inform us about ratios of both diapause phenotypes along latitudinal and altitudinal clines. This information can subsequently be implemented in phenology models (Baier et al. 2007; Jakoby et al. 2019; Jönsson et al. 2009) to estimate voltinism patterns across populations in fine resolution. Finally, combinations of various environmental factors (photoperiod, temperature, phloem quality) can contribute to a complex regulation of reproductive diapause in *I. typographus*. These combinations should be in focus of future research to get a comprehensive understanding of this developmental pathway.

## Authors’ contribution

M.S., N.D., A.S. and C.S. designed experiments. M.S. and N.D. performed experiments. M.S., N.D. and G.J.R. analysed data. All authors contributed in writing and revising the manuscript.

## Supplementary Information

Below is the link to the electronic supplementary material.

Supplementary file1 (XLSX 11 kb)

Supplementary file2 (XLSX 12 kb)

Supplementary file3 (XLSX 12 kb)

Supplementary file4 (XLSX 31 kb)

## Data Availability

All data are provided in the electronic supplementary material (File: Supplementary Information 4).
